# Differences in the Clinical Characteristics of Campylobacteriosis between Immunocompromised and Immunocompetent Patients: a Single-Center Retrospective Cohort Study in Japan

**DOI:** 10.1093/ofid/ofaf406

**Published:** 2025-07-08

**Authors:** Koh Shinohara, Yusuke Tsuda, Yasuhiro Tsuchido, Masaki Yamamoto, Yasufumi Matsumura, Miki Nagao

**Affiliations:** Department of Clinical Laboratory Medicine, Kyoto University Graduate School of Medicine, Kyoto, Japan; Department of Clinical Laboratory Medicine, Kyoto University Graduate School of Medicine, Kyoto, Japan; Department of Clinical Laboratory Medicine, Kyoto University Graduate School of Medicine, Kyoto, Japan; Department of Clinical Laboratory Medicine, Kyoto University Graduate School of Medicine, Kyoto, Japan; Department of Clinical Laboratory Medicine, Kyoto University Graduate School of Medicine, Kyoto, Japan; Department of Clinical Laboratory Medicine, Kyoto University Graduate School of Medicine, Kyoto, Japan

**Keywords:** *Campylobacter*, campylobacteriosis, foodborne infection, immunocompromised hosts

## Abstract

**Background:**

Despite growing concerns about the invasiveness of *Campylobacter* infections in patients with comorbidities, the clinical features of campylobacteriosis in immunocompromised hosts remain unclear.

**Methods:**

We conducted a retrospective cohort study on campylobacteriosis to investigate the clinical features of patients with immunosuppressive conditions in a university hospital in Japan between 2010 and 2023. The patients were classified into immunocompetent and immunocompromised groups. The clinical characteristics of the disease were compared between the groups.

**Results:**

In total, 200 patients were included: 126 in the immunocompetent group and 74 in the immunocompromised group. Patients in the immunocompromised group were significantly associated with bacteremia, especially hematopoietic stem cell transplant recipients, those with hematological malignancies, and those who received chemotherapy or steroids. Among cases with enteritis, compared with the immunocompetent group, the immunocompromised group was more likely to lack bloody stool. Prolonged duration of diarrhea over 30 days was observed in 6% of cases with diarrhea and was associated with hematological malignancy, receipt of chemotherapy, low immunoglobulin G level, and the use of rituximab. Concomitant infection with cytomegalovirus was commonly seen among cases with a prolonged duration of diarrhea.

**Conclusions:**

Our findings indicate that the clinical presentation of campylobacteriosis is affected by the patients’ immune status.

*Campylobacter* is a major cause of foodborne bacterial gastroenteritis worldwide [1]. Recently, an increasing number of *Campylobacter* invasive infections, typically accompanied by bacteremia, have been reported in multiple studies, which revealed that bacteremia mainly occurs in elderly patients with comorbidities, including immunocompromised conditions [2–7]. However, most studies were case series of bacteremia, and the remaining studies focused on the differences in patients’ backgrounds and clinical features between bacteremic and nonbacteremic campylobacteriosis. Although some previous studies have focused on the clinical features of campylobacteriosis in patients with specific immunosuppressive conditions, such as people with HIV and hematopoietic stem cell and solid transplant recipients [8–14], most of these were case series and did not compare the differences in the clinical characteristics of campylobacteriosis between immunocompromised hosts and immunocompetent hosts. Thus, despite growing attention to the importance of campylobacteriosis as an invasive infection among immunocompromised hosts, there have been limited data regarding the clinical characteristics of campylobacteriosis among immunocompromised patients compared with immunocompetent patients.

Understanding the variations in the presentation of campylobacteriosis in immunocompromised hosts could lead to earlier diagnosis and prompt, appropriate management. This study aimed to describe the detailed clinical features of *Campylobacter* infections in immunocompromised patients in comparison with immunocompetent patients.

## METHODS

### Study Population, Design, and Ethics

This single-center retrospective cohort study was conducted at Kyoto University Hospital, a university hospital in Kyoto, Japan, which has a capacity of 1131 beds. We included all patients with *Campylobacter* infections, defined by the identification of *Campylobacter* spp. in clinical samples (blood, feces, or other site culture) of patients, over a 14-year period (January 1, 2010–December 31, 2023). The molecular testing, including multiplex polymerase chain reaction (PCR) panels for gastrointestinal infections and antigen tests for *Campylobacter* spp., is not approved in Japan and not included in the identifying methods in this study. This study was approved by the Ethics Committee of Kyoto University Graduate School and Faculty of Medicine (R4499). This study was conducted in accordance with the principles of the Declaration of Helsinki. The requirement for informed consent was waived because of the retrospective nature of the study.

### Data Collection and Microbiological Analysis

We retrospectively collected clinical data regarding demographic and baseline characteristics and underlying conditions, clinical signs and symptoms, antibiotic treatment, and outcomes through medical chart review. When a patient had multiple episodes of *Campylobacter* infection, demographic and baseline characteristics at the time of the first episode were included. Laboratory results were also obtained. In microbiological diagnosis, until 2015, strains were isolated to the species level by biochemical tests, and then by matrix-assisted laser desorption/time-of-flight ionization mass spectrometry (Microflex; Bruker Daltonics, Bremen, Germany) since 2016. Antimicrobial susceptibility test results to macrolides, tetracyclines, fluoroquinolones, ampicillin, and meropenem were collected when tested. Antimicrobial susceptibility testing was interpreted according to the recommendations of the Clinical and Laboratory Standards Institute (CLSI) M45 (third edition) for *C. jejuni* and *C. coli* [15]. When CLSI breakpoints were not defined, the criteria of the National Antimicrobial Resistance Monitoring System (NARMS) or recommendations from the Antibiogram Committee of the French Society of Microbiology and European Committee on Antimicrobial Susceptibility Testing were used [16, 17]. For non-*jejuni* or -*coli Campylobacter* spp., the criteria for *C. jejuni* were applied.

### Definitions

Immunocompromised patients were defined as having any of the following conditions associated with immunosuppression: hematological malignancy, solid organ cancer, receipt of hematopoietic stem cell or solid organ transplantation, liver cirrhosis, HIV infections, connective tissue diseases, primary immunodeficiency, receipt of cytotoxic chemotherapy, steroids, immunosuppressants, biologics or small molecules within the previous 3 months, and B-cell-depleting therapy within the previous year. In patients with inflammatory bowel diseases (IBDs), those with 5-aminosalicylic acid treatment or no treatment were not considered the immunocompromised group. Serum immunoglobulin (Ig) G levels within 1 month before onset or during disease were collected. We defined serum IgG levels <500 mg/dL as low serum IgG levels. Bacteremia was defined by *Campylobacter* spp. isolation from blood culture. Diarrhea was defined as the passage of 3 or more loose or liquid stools per day. Enteritis was defined as if a case had diarrhea and positive stool culture or blood culture with *Campylobacter* spp. We classified the types of infections into 5 categories: (1) enteritis, (2) bacteremia with enteritis, (3) bacteremia without enteritis, (4) other, and (5) asymptomatic. Sepsis was diagnosed if the patient had ≥2 of the following criteria (qSOFA score): respiratory rate ≥22/min, altered mentation, or systolic blood pressure ≤100 mmHg [7, 18]. Suspected consumption history was defined as follows: eating raw or undercooked meat or raw eggs or visiting *yakiniku* or *yakitori* restaurants within 7 days before onset. *Yakiniku* restaurants are Korean/Japanese-style barbeque restaurants where people eat self-grilled beef, pork, and chicken that can be undercooked, and some of them serve raw or seared beef. *Yakitori* restaurants are Japanese-style restaurants that serve raw or seared chicken in addition to grilled chicken. Antimicrobial treatment was considered appropriate if the strain was susceptible to ≥1 of the administered antibiotics. *Campylobacter* spp. are naturally resistant to cephalosporins, piperacillin, and piperacillin/tazobactam [5, 19], and these antibiotics were considered inappropriate.

### Data Analysis

We used the chi-square test and Fisher exact test to compare categorical variables, as appropriate, and the Mann-Whitney *U* test to analyze continuous, nonparametric data; we considered a 2-sided *P* < .05 statistically significant. We performed all statistical analyses using R, version 4.4.0 (The R Foundation for Statistical Computing; https://www.r-project.org).

## RESULTS

During the study period, 210 *Campylobacter* infection episodes in 200 patients were identified and included in the study (Supplementary Figure 1). The number of cases increased before 2020, and a temporal fall was observed during 2020–2022; the number then increased again in 2023 (Supplementary Figure 2). The number of episodes peaked during summer (Supplementary Figure 3).

### Differences of Baseline and Clinical Characteristics of Campylobacteriosis Between the Immunocompetent and Immunocompromised Groups

The baseline patient characteristics are summarized in Table 1. There were 126 cases (63%) in the immunocompetent group and 74 (37%) in the immunocompromised group. *C. jejuni* (n = 172) was the most frequently identified species regardless of the disease type, whereas *C. coli* (n = 16) was identified only in enteritis or asymptomatic cases, and 3 of 4 cases with *C. fetus* were accompanied by bacteremia. We compared the clinical characteristics of campylobacteriosis between the immunocompetent and immunocompromised groups (Table 2). The median age (interquartile range [IQR]) was higher in patients in the immunocompromised group (54 [26–69] years) than in patients in the immunocompetent group (23 [19–43]; *P* < .001) (Supplementary Figure 4). Suspected consumption histories were reported in 49 (25%) of 200 cases; these numbers were similar between the groups. Cases in the immunocompromised group were significantly associated with bacteremia compared with those in the immunocompetent group (26% vs 9%; odds ratio [OR], 3.61; 95% CI, 1.61–8.11; *P* = .002), and bacteremia without enteritis was significantly more frequent in the immunocompromised group than in the immunocompetent group (16% vs 1%; OR, 24.19; 95% CI, 3.08–190.31; *P* < .001). Among the underlying conditions associated with immunocompromised status, history of hemopoietic stem cell transplantation (HSCT; 5/8 [63%] cases; OR, 17.42; 95% CI, 3.66–82.86; *P* < .001), hematological malignancy (7/14 [50%] cases; OR, 10.45; 95% CI, 3.10–35.29; *P* < .001), receipt of cytotoxic chemotherapy (7/17 [41%] cases; OR, 7.31; 95% CI, 2.32−23.04; *P* = .001), solid organ tumor (4/13 [31%] cases; OR, 4.65; 95% CI, 1.23–17.57; *P* = .035), and steroid use (8/31 [26%] cases; OR, 3.64; 95% CI, 1.32–10.03; *P* = .026) were significantly associated with bacteremia, compared with the immunocompetent group (11/126 [9%] cases). Cases of *C. fetus* infection were exclusively observed in the immunocompromised group. Seven episodes (3%) were complicated by sepsis, and 3 of these (1%) progressed to septic shock. All patients with sepsis were aged 80 years or older, and 3 were in the immunocompromised group.

**Table 1.** Baseline Characteristics of Patients With *Campylobacter* Infections, Kyoto University Hospital, Kyoto, Japan, 2011–2023 (n = 200)

Variables	No. (%)
Age, median [IQR], y	29 [21–60]
Sex	…
Male	119 (59)
Female	81 (41)
Group/underlying conditions	…
Immunocompetent group	126 (63)
Immunocompromised group	74 (37)
Solid organ tumor	13 (7)
Hematologic malignancy	14 (7)
HSCT	8 (4)
SOT	12 (6)
Cirrhosis	4 (2)
HIV	2 (1)
Collagen diseases	17 (9)
IBD^a^	11 (6)
Receipt of cytotoxic chemotherapy within 3 mo	17 (9)
Immunosuppressant use	27 (14)
Steroid use	30 (15)
Rituximab use within 6 mo	5 (3)
Type of infections	…
Enteritis	171 (86)
Bacteremia with enteritis	17 (9)
Bacteremia without enteritis	13 (7)
Other^b^	6 (3)
Asymptomatic	3 (2)
Isolated species	…
*C. jejuni*	172 (86)
*C. coli*	16 (8)
*C. fetus*	4 (2)
*C. ureolyticus*	3 (2)
*C. curvus*	1 (1)
*C. concisus*	1 (1)
*C. upsaliensis*	1 (1)
*Campylobacter* spp.	2 (1)

Abbreviations: IBD, inflammatory bowel disease; IL, interleukin; IQR, interquartile range; HSCT, hemopoietic stem cell transplantation; SOT, solid organ transplantation.

^a^Details of IBD treatment included: mesalazine alone 12, mesalazine + azathioprine 3, mesalazine + azathioprine + infliximab 2, mercaptopurine alone, mesalazine + mercaptopurine, mesalazine + infliximab (anti–tumor necrosis factor–α monoclonal antibody), mesalazine + mercaptoprine + infliximab, azathioprine + prednisolone, ustekinumab (anti-IL12/23 p40 monoclonal antibody), 1 for each.

^b^Urinary tract infections, 3; subcutaneous abscess, 3.

**Table 2.** Comparison of the Clinical Features of the Cases With *Campylobacter* Infections According to Host Immune Status, Kyoto University Hospital, Kyoto, Japan, 2011–2023 (n = 200)

Variables	Immunocompetent Group (n = 126)	Immunocompromised Group (n = 74)	Odds Ratio (95% CI)	*P* Value
Age, median [IQR], y	23 [19–43]	54 [26–69]	…	<.001
Male	75 (60)	44 (59)	1.00 (0.56–1.79)	.993
Suspected consumption history	33 (26)	16 (22)	0.78 (0.39–1.53)	.468
Consumption of raw meat or egg	21 (17)	11 (15)	0.89 (0.40–1.96)	.767
Type of infection	…	…	…	
Enteritis	108 (86)	53 (72)	0.42 (0.21–0.86)	.015
Bacteremia with enteritis	10 (8)	7 (9)	1.21 (0.44–3.33)	.794
Bacteremia without enteritis	1 (1)	12 (16)	24.19 (3.08–190.31)	<.001
Other	4 (3)	2 (3)	0.85 (0.15–4.74)	1
Asymptomatic	3 (2)	0	0 (0–inf)	.297
Bacteremia	11 (9)	19 (26)	3.61 (1.61–8.11)	.001
Isolated species	…	…	…	
*C. jejuni*	108 (86)	64 (86)	0.94 (0.41–2.16)	.879
*C. coli*	12 (10)	4 (5)	0.54 (0.16–1.75)	.420
*C. fetus*	0	4 (5)	Inf	.018
*C. ureolyticus*	3 (2)	0	0 (0–inf)	.297
*C. curvus*	0	1 (1)	Inf	.368
*C. concisus*	1 (1)	0	0 (0–inf)	1
*C. upsaliensis*	0	1 (1)	Inf	.368
*Campylobacte*r spp.	2 (2)	0	0	.531
Concurrent infection	5 (4)	14 (19)	5.65 (1.94–16.41)	<.001
CMV enteritis	1 (1)	6 ()	11.03 (1.30–93.51)	.011
*Clostridioides difficile* infection	0	2 (3)	Inf	.136
Bacteremia from Enterobacterales	1 (1)	2 (3)	3.47 (0.31–38.97)	.556
Others: polymicrobial infections at the focus	2 (2)	2 (3)	1.72 (0.23–12.49)	1
Other: concurrent infection outside the focus	1 (1)	4 (5)	7.14 (0.78–65.16)	.062
Hospitalization	47 (37)	52 (70)	3.92 (2.12–7.26)	<.001
Length of stay, median [IQR], d	7 [5–13]	11 [7–29]	…	.016
Any antimicrobial administration	62 (49)	53 (70)	2.61 (1.41–4.81)	.020
Appropriate antimicrobial treatment	33 (26)	41 (55)	3.50 (1.91–6.42)	<.001
Onset to appropriate treatment, median [IQR], d	4 [3–7]	4 [2–7]	…	.862
Duration of appropriate treatment, median [IQR], d	5 [3–7]	7 [5–14]	…	.017
Outcome	…	…	…	
Admission to ICU	0	3 (4)	Inf	.049
Mechanical ventilation	0	1 (1)	Inf	.368
Catecholamine use	1 (1)	1 (1)	1.71 (0.11–27.79)	1
90-d Mortality	1/75 (1)	1/69 (1)	1.09 (0.07–17.74)	1

Abbreviations: CMV, cytomegalovirus; ICU, intensive care unit.

Antimicrobial treatments were administered to 58% of all patients, with a higher frequency in the immunocompromised group (70% vs 49%; *P* = .020). However, only 37% of all patients received appropriate antibiotics, 55% in the immunocompromised group. Among the cases who presented with bacteremia without enteritis, empiric antibiotic treatment was appropriate in 5 of 13 cases (38%), and 3 of these cases involved the use of carbapenems. The median time from symptom onset to administration of appropriate treatment was 4 days, with no significant difference between the groups. In the immunocompromised group, appropriate antibiotics were more often administered after culture results were reported compared with the immunocompetent group (11/74 [15%] vs 5/126 [4%]; *P* = .012). Nine of these 16 cases—including 6 immunocompromised patients—had experienced prolonged fever or diarrhea, while 7 cases were clinically improving or had already improved by the time antibiotics were administered. Although 5 of the 16 cases had suspected consumption histories, the histories were taken after reporting the culture results.

Concurrent infections were observed significantly more frequently in the immunocompromised group (14/74 [19%] cases) than the immunocompetent group (4/126 [4%]; *P* < .001). Cytomegalovirus (CMV) enteritis was the most common concurrent infection (7 cases); 6 of these cases occurred in the immunocompromised group, and the other 1 case occurred in a patient with IBD. In other concurrent infections, *Clostridioides difficile* infection (2 cases) was only observed in the immunocompromised group, and concurrent infections outside the focus of *Campylobacter* infection were more commonly seen in the immunocompromised group.

We then compared the detailed clinical features of enteritis, regardless of bacteremia, according to immune status (Table 3). There were 118 cases in the immunocompetent group, 50 in the immunocompromised group. The median age was higher in the immunocompromised group (Supplementary Figure 4). At presentation, bloody stool was less common in the immunocompromised group than in the immunocompetent group (OR, 0.33; 95% CI, 0.13–0.79; *P* = .014). The proportion of patients who obtained blood cultures and the positive rates of blood cultures were not significantly different between the immunocompromised and immunocompetent groups. Compared with the immunocompetent group, appropriate antibiotics were administered significantly more frequently in the immunocompromised group (OR, 2.46; 95% CI, 1.28–4.71; *P* = .006). Patients in the immunocompromised group were hospitalized more frequently than those in the immunocompetent group (OR, 3.13; 95% CI, 1.64–5.97; *P* < .001).

**Table 3.** Comparison of the Clinical Characteristics of the Cases With Enteritis According to Immune Status, Kyoto University Hospital, Kyoto, Japan, 2011–2023 (n = 178)

Variables	Immunocompetent Group (n = 118)	Immunocompromised Group (n = 60)	Odds Ratio (95% CI)	*P* Value, Immunocompetent vs Immunocompromised
Age, median [IQR], y	23 [19–39]	51 [25–68]	…	<.001
Male	71 (60)	35 (58)	0.93 (0.49–1.74)	.813
Symptoms at presentation	…	…	…	…
Fever	82 (69)	45 (75)	1.32 (0.65–2.66)	.442
Abdominal pain	77 (65)	33 (55)	0.65 (0.35–1.23)	.183
Bloody stool	34 (29)	7 (12)	0.33 (0.13–0.79)	.014
Vomiting	11 (9)	4 (7)	0.69 (0.21–2.28)	.547
Max No. of diarrhea episodes/d	…	…	…	…
>10 times	32/80 (40)	20/44 (45)	1.25 (0.59–2.63)	.574
3–10 times	48/80 (60)	24/44 (55)	…	…
Examination	…	…	…	…
Abdominal CT	39 (33)	23 (38)	1.26 (0.66–2.40)	.484
Colonoscopy	18 (15)	10 (17)	1.11 (0.48–2.58)	.807
Blood culture obtained	46 (39)	28 (47)	1.37 (0.73–2.57)	.325
Bacteremia	10/46 (22)	7/28 (25)	1.2 (0.40–3.62)	.781
Hospitalization	42 (36)	38 (63)	3.13 (1.64–5.97)	< .001
Hospital d	6 [5–10]	8 [5–18]	…	.111
Administration of antibiotics, any	59 (50)	39 (65)	1.86 (0.98–3.53)	.057
Administration of appropriate antibiotics	31 (26)	28 (47)	2.46 (1.28–4.71)	.006
Days from onset to appropriate antibiotics	4 [3–7]	4 [2–7]	…	.812
Isolated species	…	…	…	…
*C. jejuni*	105 (89)	55 (92)	1.36 (0.46–4.02)	.793
Non-*jejuni Campylobacter*	13 (11)	5 (8)	…	…
Outcome	…	…	…	…
90-d all-cause mortality	0/69	1/59 (2)	Inf	.461
Reinfection/relapse	3/67 (4)	3/57 (5)	1.19 (0.23–6.11)	1
>30 d from onset to resolution of diarrhea	0/71	7/54 (13)	Inf	.002

Abbreviation: CT, computed tomography.

### Antimicrobial Susceptibility

Among the 210 *Campylobacter* isolates, resistance rates to macrolides, tetracyclines, ampicillin, and fluoroquinolones were 3% (5/199), 18% (36/199), 38% (75/199), and 58% (116/199), respectively. *C. fetus* was more resistant to macrolides than *C. jejuni* (2/8 [25%] vs 2/172 [1.2%]; *P* = .010), and *C. coli* was more resistant to ampicillin than *C. jejuni* (11/16 [69%] vs 62/172 [36%]; *P* = .015). Fluoroquinolone resistance rates were significantly lower in the second half (2017–2023, 58/112 [52%]) of the study period, compared with the first half (2010–2016, 58/87 [67%]; *P* = .035), and macrolide resistance (5 isolates) was observed only in the second half. Resistance rates to each antimicrobial were not significantly different between the immunocompetent and immunocompromised groups, whereas 4 of 5 (80%) macrolide-resistant isolates were isolated from the immunocompromised group (Supplementary Table 1).

### Campylobacteriosis in Hemopoietic Stem Cell Transplant Recipients and Solid Organ Transplant Recipients

There were 8 cases with a history of HSCT, including 1 patient who had a history of both HSCT and solid organ transplant (SOT), who presented with enteritis without bacteremia (Table 4).

**Table 4.** Campylobacteriosis in Hematopoietic Stem Cell Recipients, Kyoto University Hospital, Kyoto, Japan, 2011–2023

No.	Age/Sex	Indication and Type of Transplant	Duration From Transplant to Onset, mo	GvHD/Neutropenia	Immunosuppressants	Type of Infection	Isolated Species	Concomitant Infection	Treatment	New-Onset or Exacerbation of GvHD After Infection
1	54F	Myelofibrosis, allo-PBSCT	10	GvHD (intestinal)	PSL (20 mg/d)	Bacteremia without enteritis	*C. jejuni*	Disseminated aspergillosis	FEP (IV, 4 d) −> MEM (IV, 13 d) −> CRO (IV, 3 d)	-
2	48F	MDS, BMT	26	GvHD (skin)	PSL (30 mg/d) + Tac	Bacteremia without enteritis	*C. jejuni*	CMV enteritis	TZP (IV, 3 d) −> TZP/AZM (IV, 3d) −> TZP/LVX (IV, 7 d)	-
3	47M	AA/MDS, CBT	37	GvHD (skin/liver/eye)	PSL (2 mg/d)	Bacteremia without enteritis	*C. jejuni*	-	CRO (IV, 3 d) −> AZM (PO, 3 d) −> AZM (PO, 3 d)	-
4	58F	DLBCL, CBT	26	Neutropenic^a^	Flu/Cy^b^	Bacteremia without enteritis	*C. jejuni*	-	FEP (IV, 3 d) −> TZP (IV, 2 d) −> MEM (IV, 14 d)/AZM (PO, 3 d)	-
5	27M	Ewing sarcoma, auto-PBSCT	0 (day 4)	Neutropenic	Flu/Bu^c^	Enteritis	*C. coli*	-	FEP (IV, 2 d) −> MEM (IV, 5 d) −> CRO (IV, 7 d)	-
6	58M	ALL, allo-PBSCT	4	None	Tac	Enteritis	*C. jejuni*	-	-	New-onset GvHD (intestinal, concomitant)
7	31F	LBL, CBT	0 (day 3)	Neutropenic	Cy/TBI^d^	Bacteremia with enteritis	*C. jejuni*	CMV retinitis	TZP (IV, 2 d) −> FEP (IV, 4 d) −> MEM (IV, 18 d) −> CRO (IV, 2 d) −> TZP (IV, 6 d) −> FEP (IV, 7 d)	New-onset GvHD (intestinal, 2 wk after infection)
8	24M	ALL/BO, BMT/lung, living donor	173 (BMT)/20 (lung)	None	Tac + MMF + PSL (5 mg/d)	Enteritis	*C. jejuni*	-	-	-

Abbreviations: -, none/not available; AA, aplastic anemia; ALL, acute lymphoblastic leukemia; AZA, azathioprine; AZM, azithromycin; BMT, bone marrow transplant; BO, bronchiolitis obliterans; CAR-T, chimeric antigen receptor T cell; CBT, cord blood transplant; CMV, cytomegalovirus; CRO, ceftriaxone; Cy/TBI, cyclophosphamide and total body irradiation; DLBCL, diffuse large B-cell lymphoma; FEP, cefepime; Flu/Bu, fludarabine/busulfan; Flu/Cy, fludarabine/cyclophosphamide; GvHD, graft-vs-host disease; HSCT, hematopoietic stem cell transplant; LBL, lymphoblastic lymphoma; LVX, levofloxacin; MDS, myelodysplastic syndrome; MEM, meropenem; MMF, mycophenolate mofetil; MZR, mizoribine; NOS, not otherwise specified; PBSCT, peripheral blood stem cell transplant; PSL, prednisolone; SOT, solid organ transplant; Tac, tacrolimus; TZP, piperacillin/tazobactam.

^a^This case onset occurred under neutropenia during CAR-T therapy for relapsed DLBCL.

^b^As conditioning regimen for CAR-T therapy, 6 days before onset.

^c^As conditioning regimen for auto-PBSCT, 11 days before onset.

^d^As conditioning regimen for CBT, 4 days before onset.

In HSCT recipients, transplantation types were allogeneic in 7 cases and autologous in 1 case. Six of the 8 patients (75%) had 1 of the following factors: (i) prior diagnosis of graft-vs-host disease (GvHD) with immunosuppressive treatment (3 cases) or (ii) neutropenic status due to high-dose chemotherapy (3 cases). Among the 8 HSCT cases, 5 (63%) had bacteremia, 4 without enteritis. All patients with GvHD presented with bacteremia without enteritis. Concomitant infections were observed in 3 of 8 cases (38%): CMV enteritis, CMV retinitis, and microbiologically confirmed disseminated aspergillosis, 1 case each.

Among SOT recipients, there were 8 kidney, 2 liver, and 1 lung transplant recipients (Supplementary Table 2). Ten patients received grafts from living donors, 6 of whom were ABO-incompatible. All cases occurred >1 year after transplantation, and 8 (73%) occurred >5 years after transplantation. Among the cases, 3 (27%) had bacteremia and 2 had bacteremia without enteritis. Although the 3 cases with bacteremia were treated with appropriate antimicrobials, only 1 of 8 cases (13%) with enteritis was treated with appropriate antimicrobials. Although 1 case was diagnosed with chronic active antibody-mediated rejection via a scheduled kidney biopsy—performed 4 months after the detection of donor-specific antibodies and coinciding with the isolation of *C. jejuni* from a stool culture due to diarrhea that began a few days prior—the clinical course suggested that the campylobacteriosis did not trigger the rejection.

### Clinical Features of the Campylobacteriosis Cases With Prolonged Duration of Diarrhea Over 30 Days and the Cases With Multiple Episodes

Among the 188 cases of enteritis, regardless of bacteremia, the duration of diarrhea from onset to resolution was followed in 125 cases (Table 5). Seven (6%) patients required >30 days from symptom onset to resolution. All 7 cases were in the immunocompromised group, and 4 were treated with a regimen including rituximab for malignant lymphoma or primary macroglobulinemia. Three of the 7 cases (43%) had concurrent CMV enteritis, all pathologically confirmed. Antimicrobial treatments were administered in 4 of the 7 cases, whereas only 2 received antimicrobial agents susceptible to the isolates. In these 2 cases, with the administration of appropriate antimicrobial treatment, the diarrhea was prolonged over 1 month after the administration of susceptible antibiotics, and 1 case was resolved after secondary treatment. One of the remaining 3 patients without antibiotic administration was treated for concurrent CMV enteritis, which was refractory and resolved after ∼7 months. None of the patients underwent a follow-up stool culture for diarrheal symptoms. Significant risk factors associated with prolonged duration of diarrhea symptoms for >30 days were underlying hematological malignancy (OR, 19.87; 95% CI, 2.80–164.52; *P* = .001), receipt of cytotoxic chemotherapy within 3 months of onset (OR, 7.85; 95% CI, 1.01–54.16; *P* = .024), receipt of rituximab within a year of onset (OR, 156.00; 95% CI, 16.60–1346.42; *P* < .001), and low serum IgG level (IgG <500 mg/dL) within a month before onset or during the disease (OR, 38.19; 95% CI, 2.53–2493.1; *P* = .002) (Supplementary Table 3). All 3 cases with concurrent CMV enteritis had low serum IgG levels. Two of the 4 cases with prolonged diarrheal symptoms and low serum IgG levels received intravenous immunoglobulin during the disease; however, diarrheal symptoms continued for 14 days after administration in both cases.

**Table 5.** Cases With Prolonged Duration of Diarrhea Over 30 Days, Kyoto University Hospital, Kyoto, Japan, 2011–2023

No.	Age/Sex	Group/Underlying Conditions	Immunosuppressive Therapy	Rituximab Use	Serum IgG Level, mg/dL	Isolated Organisms	AMR	Concomitant Infections	Treatment	Onset to Initiation of Antibiotics, d^d^	Onset to Resolution of Diarrhea, d	Multiple Campylobacteriosis Episodes
1	71F	Immunocompromised/RA, MTX-LPD	Rituximab	Yes	NA	*C. jejuni*	-	-	-	-	77	No
2	25M	Immunocompromised/PID	PSL (5 mg/d) + canakinumab^a^	No	1240	*C. jejuni*	-	-	LVX (PO, 3 d)	55	68	Yes (enteritis in 84 mo)
3	50M	Immunocompromised/primary macroglobulinemia	BR	Yes	148	*C. jejuni*	-	CMV enteritis	-	-	225	No
4	71F	Immunocompromised/FL	BR	Yes	405	*C. jejuni*	-	CMV enteritis	LVX (PO, 5 d)/LVX (PO, 10 d)	28/63	88	No
5	74M	Immunocompromised/ML, colon cancer	CP-R	Yes	206	*C. jejuni*	Macrolides/FQs	CMV enteritis	AZM (PO, 10 d)	(23)	41	Yes (bacteremia in 4 mo)
6	63M	Immunocompromised/dermatomyositis	PSL (10 mg/d) + Tac + MMF	No	254	*C. jejuni*	Tetracyclines/FQs	Pneumonia without identified pathogen	LVX (PO, 15 d)	(34)	48	No
7	23F	Immunocompromised/UC	Ustekinumab^b^	No	NA	*C. jejuni*	NA^c^	-	-	-	89	No

Abbreviations: -, none; AMR, antimicrobial resistance; AZM, azithromycin; BR, bendamustine plus rituximab; CMV, cytomegalovirus; CP-R, cyclophosphamide/prednisone plus rituximab; FL, follicular lymphoma; FQs, fluoroquinolones; IBD, inflammatory bowel disease; IL, interleukin; LVX, levofloxacin; ML, malignant lymphoma; MMF, mycophenolate mofetil; MTX-LPD, methotrexate-associated lymphoproliferative disorders; NA, not available; PID, primary immunodeficiency; PSL, prednisolone; RA, rheumatoid arthritis; Tac, tacrolimus; UC, ulcerative colitis.

^a^Anti-IL-1β monoclonal antibody.

^b^Anti-IL12/23 p40 monoclonal antibody.

^c^Results of antimicrobial susceptibility test were not available due to growth failure.

^d^If nonsusceptible antimicrobials were administered, days were represented as numbers in parentheses.

### Cases With Multiple Episodes of Campylobacteriosis

Seven (4%) patients experienced multiple episodes of campylobacteriosis during the study period (Supplementary Table 4). In 2 (29%) cases, the causative organisms were different between episodes. In the other 5 cases, the same species were identified throughout the episodes; however, the intervals of the episodes were at least 4 months. One patient experienced 5 episodes of *C. fetus* bacteremia, received prednisolone for vasculitis and autoimmune hepatitis, and was implanted with a cardiac pacemaker.

## DISCUSSION

In this study, we revealed detailed clinical presentations of campylobacteriosis according to immune status. Some previous studies have focused on the differences between bacteremic and nonbacteremic campylobacteriosis cases [20, 21]; however, few studies have assessed differences in the detailed clinical presentations of campylobacteriosis according to immune status. Patients in the immunocompromised group were more likely to present with bacteremia without enteritis, especially those at high risk among hematopoietic stem cell transplant recipients. In the immunocompromised group, enteritis was less likely to be accompanied by abdominal pain and bloody stool, which might resemble sepsis with nonspecific diarrhea rather than bacterial enteritis. This might be associated with the higher rates of appropriate antibiotic administration in this group. Sepsis was observed in only 3% of patients, and the diseases were generally mild even in the immunocompromised group. The median duration from symptom onset to administration of appropriate antibiotics was 4 days, with no significant difference between groups. There are several possible explanations for this delay. The first is the difficulty of diagnosing campylobacteriosis in patients without diarrhea. In our study, only 38% of bacteremic patients without enteritis received appropriate empiric therapy. The second reason is the relatively long time required to report culture results. The median duration from sample collection to reporting was 5 days. In most cases, the symptoms were getting better before the results were reported. In addition, among immunocompromised hosts, multiple potential causes of diarrhea—such as CMV enteritis—make it difficult to consider campylobacteriosis as the primary cause without a clear history of suspected food consumption. Indeed, appropriate antibiotics were administered after culture results were reported in 15% of immunocompromised patients.

Campylobacteriosis is one of the most frequently encountered foodborne diseases [10, 11]. However, the detailed characteristics of campylobacteriosis in these patients remain unclear. In our study, 2 distinct clinical features of HSCT recipients appeared to be associated with campylobacteriosis. One was the underlying GvHD, and the other was neutropenic conditions with high-dose chemotherapy as conditioning regimens for HSCT or chimeric antigen receptor T cell (CAR-T) therapy. Bacteremia was commonly observed in the HSCT recipients (5 of 8 cases, including a patient with both HSCT and SOT), and interestingly, all 3 cases with underlying GvHD presented as bacteremia without enteritis, whereas 2 of 3 neutropenic HSCT cases presented with enteritis. In a previous case series of foodborne infections in HSCT recipients, 5 cases of campylobacteriosis were reported, and all cases had a history of gut GvHD or neutropenia [11], which supports our findings. Our study highlights the importance of *Campylobacter* as a cause of bacteremia in HSCT recipients and that GvHD and neutropenia might be among the risk factors for developing invasive campylobacteriosis.

*Campylobacter* is also a leading cause of bacterial foodborne infections in SOT recipients [13, 22]. In our study, bacteremia occurred in 3 of 12 (25%) campylobacteriosis cases in SOT recipients, a rate higher than that reported in nationwide studies in Switzerland (3% [3/125]) and France (5% [16/326]) [12, 13]. Although a small number of cases and the single-center nature of this study set in a university hospital would be major causes for this difference, the high proportion of living donor transplantation (91% [11/12]) and a history of rejection, including ABO-incompatible antibody-mediated rejection (2 of 3 bacteremic cases had histories of rejection), might be associated with the higher incidence of bacteremia in our study.

In our study, prolonged duration of diarrhea was observed in 6% of the enteritis cases with an identified duration of symptoms, and all the cases were in the immunocompromised group or the IBD group with receipt of immunosuppressive biological products. Low IgG levels and use of rituximab, a B-cell-depleting anti-CD20 monoclonal antibody, were significantly associated with prolonged duration of diarrhea. Previous case series and cohort studies reported prolonged or recurrent diarrheal symptoms of *Campylobacter* enteritis among patients with primary immunodeficiency with very low immunoglobulin levels, such as X-linked agammaglobulinemia and common variant immunodeficiency (CVID), which supports our results [23–26]. However, the results should be interpreted with caution because follow-up culture was not performed in our cases and concurrent CMV enteritis, observed in 3 of 7 cases, might play a key role in some cases.

Suspected consumption history, defined as a recent history of eating raw meat or eggs and visiting *yakiniku*/*yakitori* restaurants, was reported in approximately one-fourth of the cases and was not significantly different among the groups. Bacterial foodborne pathogens such as *Listeria monocytogenes*, *Salmonella*, and *Campylobacter* are more likely to cause invasive infections, with substantial morbidity and mortality in patients with immunosuppressive conditions [5, 27, 28]. A recent study in solid organ transplant recipients revealed that although the majority of recipients felt concerned about food safety, only 27% were able to recognize all risks of foodborne infection in hypothetical scenarios [22]. Education to avoid foods that are high-risk for foodborne pathogens among high-risk populations should be emphasized.

In this study, the number of campylobacteriosis cases peaked during the summer season. Previous studies have reported the same seasonal trend in campylobacteriosis in other regions [29, 30]. Considering the strong tide of the warming climate and extreme weather events associated with climate change, the incidence of campylobacteriosis is expected to increase [31], as in the cases of *Vibrio* infections [32] and *E. coli* bloodstream infections [33]. Understanding the clinical features of campylobacteriosis in immunocompromised hosts is critical to confront the upcoming increased burden of campylobacteriosis in this group.

In conclusion, we found that the clinical presentation of campylobacteriosis varied according to the underlying immune conditions of the patients. Hematological malignancy, receipt of cytotoxic chemotherapy, and HSCT were associated with bacteremia, whereas humoral immune defects, often related to rituximab use, were associated with a prolonged duration of diarrheal symptoms. Our findings underscore the importance of patient background for the presentation of campylobacteriosis.

## Supplementary Material

ofaf406_Supplementary_Data

## Supplementary Data

Supplementary materials are available at *Open Forum Infectious Diseases* online. Consisting of data provided by the authors to benefit the reader, the posted materials are not copyedited and are the sole responsibility of the authors, so questions or comments should be addressed to the corresponding author.
