# Supplementary material for: Differences in the Clinical Characteristics of Campylobacteriosis between Immunocompromised and Immunocompetent Patients: a Single-Center Retrospective Cohort Study in Japan
Source: Open Forum Infect Dis. 2025 Jul 8;12(8):ofaf406. doi: 10.1093/ofid/ofaf406 (PMC12343107; doi:10.1093/ofid/ofaf406)

Supplementary Materials

Supplementary Table 1. Antimicrobial susceptibility of the isolates, Kyoto University Hospital, Kyoto, Japan, 2011-2023 (n = 210)

	**All**	***C. jejuni****	***C. coli***	***C. fetus***	others*	**2010-2016**	**2017-2023**	**p-value**	Immunocompetent	**Immunocompromised**	**p-value**
Macrolides	5/199 (3%)	2/172 (1%)	1/16 (6%)	2/8 (25%)	0	0/87	5/112 (5%)	0.069	1/122 (1%)	4/77 (5%)	0.075
Tetracyclines	36/199 (18%)	30/172 (17%)	4/16 (25%)	2/25 (25%)	0	13/87 15%)	23/112 (21%)	0.309	24/112 (20%)	12/77 (16%)	0.466
Ampicillin	75/199 (38%)	62/172 (36%)	11/16 (69%)	0	2/3 (67%)	37/87 (43%)	38/112 (34%)	0.214	47/122 (39%)	28/77 (36%)	0.759
Fluoroquinolones	116/199 (58%)	96/172 (56%)	13/16 (81%)	4/8 (50%)	3/3 (100%)	58/87 (67%)	58/112 (52%)	0.035	46/122 (38%)	37/77 (48%)	0.149

*: Antimicrobial susceptibilities of the 6 isolates of *C. jejuni* and 5 isolates of other C*ampylobacter* spp. were not determined due to growth failure.

Supplementary Table 2. Campylobacteriosis in solid transplant recipients, Kyoto University Hospital, Kyoto, Japan, 2011-2023 (excluding a recipient with histories of HSCT and SOT)

No.	Age/Sex	Indication and type of transplant	Duration from transplant to onset, month	Rejection prior to infection	Immunosuppressants	Type of infection	Isolated species	Concomitant infection	Treatment	Newly onset or exacerbation of rejection after infection
1	25 M	Fulminant hepatitis /Liver, living donor, ABO-incompatible	131	ABO-incompatible AMR, ACR	PSL (12mg/d) + Tac+ MMF + Abatacept + Upadacitinib	Bacteremia without enteritis	*C. jejuni*	*E. coli* bacteremia	MEM (iv, 14d)	-
2	61 M	Fulminant HBV hepatitis /Liver, living donor, ABO-incompatible	84	ABO-incompatible AMR	Tac + MMF	Bacteremia without enteritis, vertebral osteomyelitis	*C. jejuni*	-	MEM (iv, 28d) -> CLR/TMP-SMX (po, 13d) -> CLR/TMP-SMX/MIN (po, 12d) -> CLR/MIN (po, 61d)	-
3	81 M	IgA nephropathy /Kidney, living donor, ABO-incompatible	63	AMR, ACR	Tac + MZR + PSL (5mg/d)	Enteritis	*C. jejuni*	-	-	-
4	74 F	Membranous nephropathy /Kidney, living donor	209	None	Tac + MMF + PSL (5mg/d)	Enteritis	*C. jejuni*	-	CMZ (iv, 12d)	-
5	41 F	IgA nephropathy /Kidney, living donor	18	None	Tac + MMF + PSL (5mg/d)	Enteritis	*C. jejuni*	-	-	-
6	25 M	Pulmonary arterial hypertension /Lung, deceased donor	37	None	Tac + MMF + PSL (5mg/d)	Enteritis	*C. jejuni*	-	LVX (po, 5d)	-
7	48 F	Lupus nephritis /Kidney, living donor	13	None	Tac + PSL (5mg/d)	Bacteremia with enteritis	*C. jejuni*	-	LVX (iv, 9d) -> AZM (po, 5d)	-
8	44 M	Chronic nephritis, NOS /Kidney, living donor	280	None	CyA + AZA + PSL (5mg/d)	Enteritis	*C. jejuni*	-	-	newly diagnosed AMR*
9	62 M	Membranoproliferative glomerulonephritis /Kidney, living donor, ABO-incompatible	101	None	Tac + MMF + PSL (5mg/d)	Enteritis	*C. jejuni*	-	-	-
10	54 F	IgA nephropathy /Kidney, living donor, ABO-incompatible	61	None	Tac + MMF + PSL (5mg/d)	Enteritis	*C. jejuni*	HSV encephalitis	-	-
11	63 F	Chronic renal failure /Kidney, living donor, ABO-incompatible	108	None	Tac + MMF + Everolimus	Enteritis	*C. jejuni*	-	-	-

Abbreviations: M, male; F, female; NOS, not otherwise specified; AMR, antibody-mediated rejection; ACR, acute cellular rejection; PSL, prednisolone; d, days; Tac, tacrolimus; MMF, mycophenolate mofetil; MZR, mizoribine; CyA, cyclosporine A; AZA, azathioprine; HSV, herpes simplex virus; -, none/not available; MEM, meropenem; CLR, clarithromycin; TMP-SMX, trimethoprim/sulfamethoxazole; MIN, minocycline; CMZ, cefmetazole; LVX, levofloxacin; AZM, azithromycin

*: This case was diagnosed as chronic active AMR by a planned kidney biopsy performed four months after the detection of de novo donor-specific antibodies. The patient had experienced diarrhea a few days before the biopsy and *C. jejuni* was isolated from stool culture.

Supplementary Table 3. Risk factors associated with prolonged duration of diarrhea in cases with enteritis, Kyoto University Hospital, Kyoto, Japan, 2011-2023

Variables	Case with prolonged duration of diarrhea, n (%), N = 7	Cases without prolonged duration of diarrhea, n (%), N = 118	p-value
Age in year, median [IQR]	63 [38 - 71]	30 [20 – 61]	0.09
Male sex	4 (57)	65 (55)	1
Solid organ tumor	1 (14)	9 (8)	0.450
Hematological malignancy	4 (57)	7 (6)	0.001
Inflammatory bowel diseases	1 (14)	23 (19)	1
Collagen disorder	2 (29)	12 (10)	0.177
Chemotherapy recipient	3 (43)	10 (8)	0.024
Immunosuppressant use	2 (29)	19 (16)	0.334
Steroid use	1 (14)	18 (15)	1
Rituximab use	4 (57)	1 (1)	< 0.001
White blood cell count at diagnosis, /mm^3, median [IQR]	5000 [4815 – 8395]	7800 [5890 – 11815], n = 115	0.178
Neutrocyte count at diagnosis, /mm^3, median [IQR]	4150 [3325 – 6918], n = 6	6100 [3860 – 9490], n = 109	0.311
Serum IgG level within 6 months prior to diagnosis, mg/dL, median [IQR]	329.5 [218 – 673.5], n = 5	1162.0 [919.5 – 1362.0], n = 27	0.002
Low serum IgG level	4/5 (80)	2/27 (7)	0.002

Abbreviations: IQR, interquartile range

Supplementary Table 4. Cases with multiple episodes of campylobacteriosis, Kyoto University Hospital, Kyoto, Japan, 2011-2023

Case/ Episod e	Ag e	Se x	Group/Underlying conditions	Isolated samples and organisms	Types of infection	Treatment	Duration between the last episode, month	Suspected consumption history
A/1	73	M	Immunocompromised/Lung cancer	stool/*C. coli*	Enteritis	-	-	-
/2	80			stool/*C. jejuni*	Enteritis	-	84	-
B/1	18	F	Immunocompetent	stool/*C. coli*	Enteritis	-	-	yakiniku
/2	19			stool/*C. jejuni*	Enteritis	-	12	-
C/1	25	M	Immunocompromised/PID	stool/*C. jejuni*	Enteritis	LVX (p.o., 3d)	-	-
/2	28			stool/*C. jejuni*	Enteritis	AZM (p.o., 3d)	47	raw egg
D/1	18	M	IBD/UC	stool/*C. jejuni*	Enteritis	-	-	-
/2	19			stool/*C. jejuni*	Enteritis	-	52	-
E/1	69	M	Immunocompromised/AIH, RPGN, PMR, SSS/CAVB, indwelling PM	blood/*C. fetus*	Bacteremia without enteritis	IPM (i.v., 14d) -> LVX (i.v., 14d) -> LVX (p.o., 42d)	-	-
/2	71			blood/*C. fetus*	Bacteremia without enteritis	-	19	-
/3	72			blood/*C. fetus*	Bacteremia without enteritis	-	10	-
/4	72			blood/*C. fetus*	Bacteremia without enteritis	AMC (p.o., 35d)	1	-
/5	73			blood/*C. fetus*	Bacteremia without enteritis	AMC (p.o., 10d) -> IPM (i.v., 1d) -> MEM (i.v., 16 d) -> AMC (p.o., 10d) -> MEM (i.v., 7d) -> AMC (p.o., 49d)	18	-
F/1	22	M	IBD/UC	stool/*C. jejuni*	Enteritis	-	-	-
/2	22			stool/*C. jejuni*	Enteritis	-	4	yakitori
G/1	74	M	Immunocompromised/ ML	stool, blood/*C. jejuni*	Bacteremia with enteritis	AZM (p.o., 3d)	-	-
/2	74			blood/*C. jejuni*	Bacteremia without enteritis	MEM + GEN (i.v., 30d)	16	raw poultry

Abbreviations: M, male; F, female; PID, primary immunodeficiency; IBD, inflammatory bowel syndrome; UC, ulcerative colitis; AIH, autoimmune hepatitis; RPGN, rapidly progressing glomerulonephritis; PMR, polymyalgia rheumatica; SSS, sick sinus syndrome; CAVB, complete atrio-ventricular block; PM, pacemaker; ML, malignant lymphoma; p.o., orally; i.v., intravenously; d, days; -, not administered/not available; LVX, levofloxacin; AZM, azithromycin; IPM, imipenem; AMC, amoxicillin/clavulanic acid; MEM, meropenem; GEN, gentamicin

Supplementary Figure 1. Flowchart of 200 patients with *Campylobacter* spp. infections in Kyoto University Hospital, Kyoto, Japan, 2010-2023

Alt text: The flowchart of the included patients into the study.

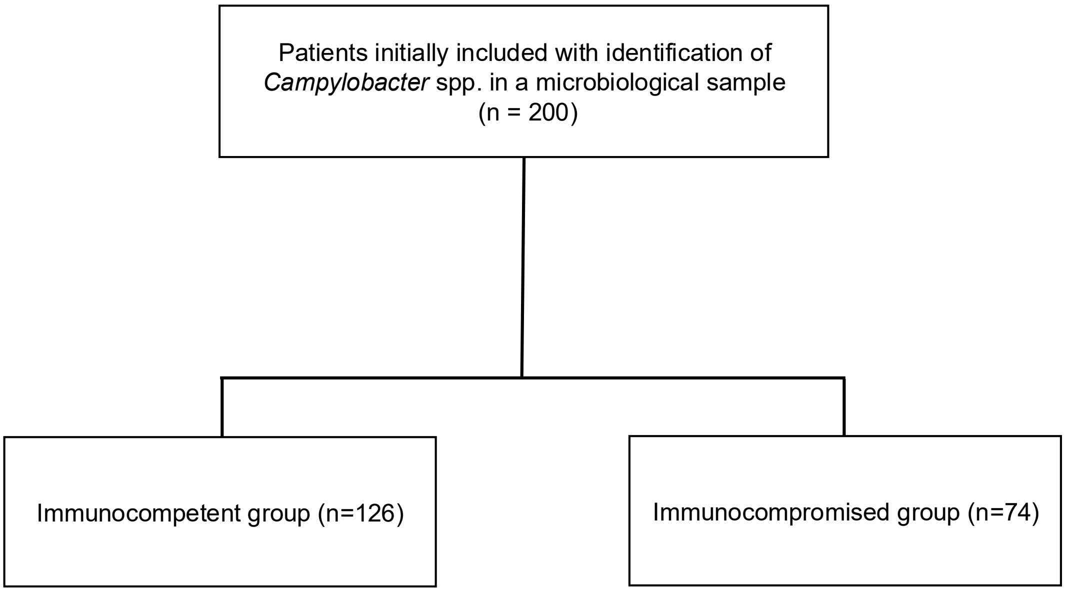

Supplementary Figure 2. Temporal distribution of the cases according to the groups in Kyoto University Hospital, Kyoto, Japan, 2010-2023 (N=200)

Alt text: The bar chart describing the temporal distributions of the cases according to the groups.

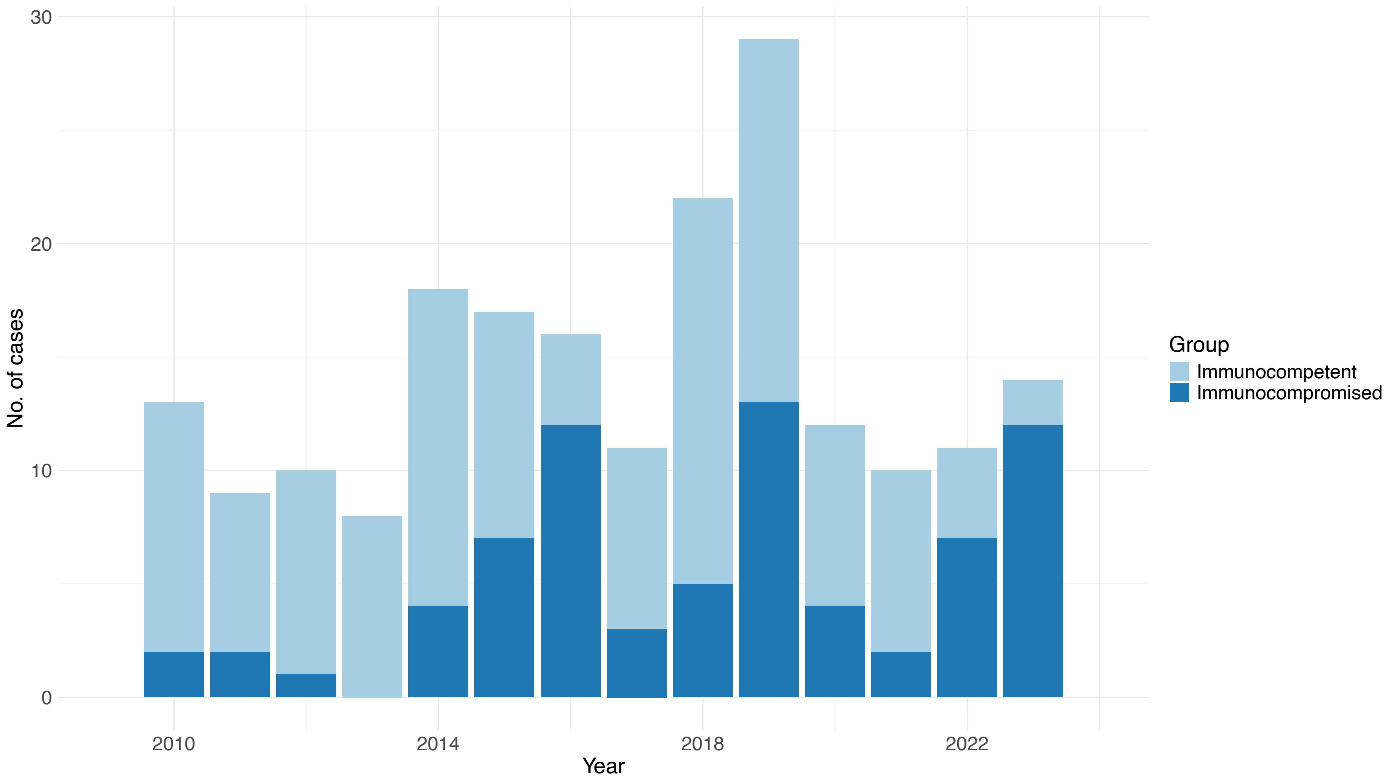

Supplementary Figure 3. Seasonal distribution of the cases in Kyoto University Hospital, Kyoto, Japan, 2010-2023 (N = 210)

Alt text: The bar chart describing the seasonal distribution of the cases, which shows its peak in the summer season.

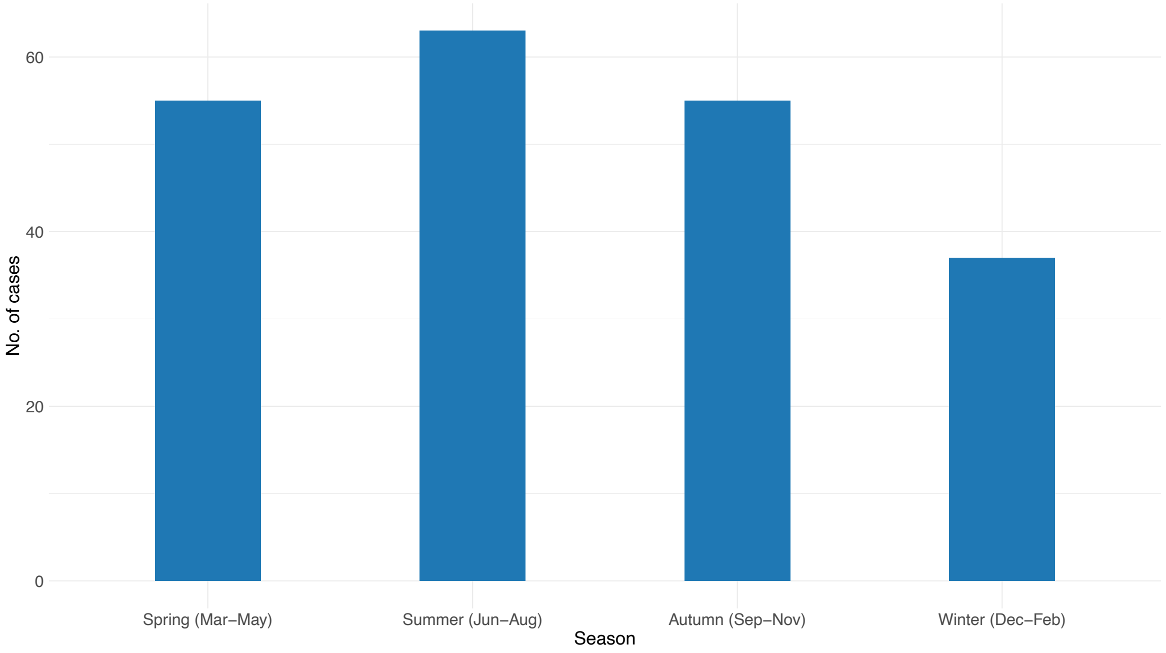

VX

Supplementary Figure 4. Age distribution of the cases with campylobacteriosis according to the types of infections and the groups, Kyoto University Hospital, Kyoto, Japan

Alt text: The histograms showing the age distribution of the case according to the types of infections and the groups.

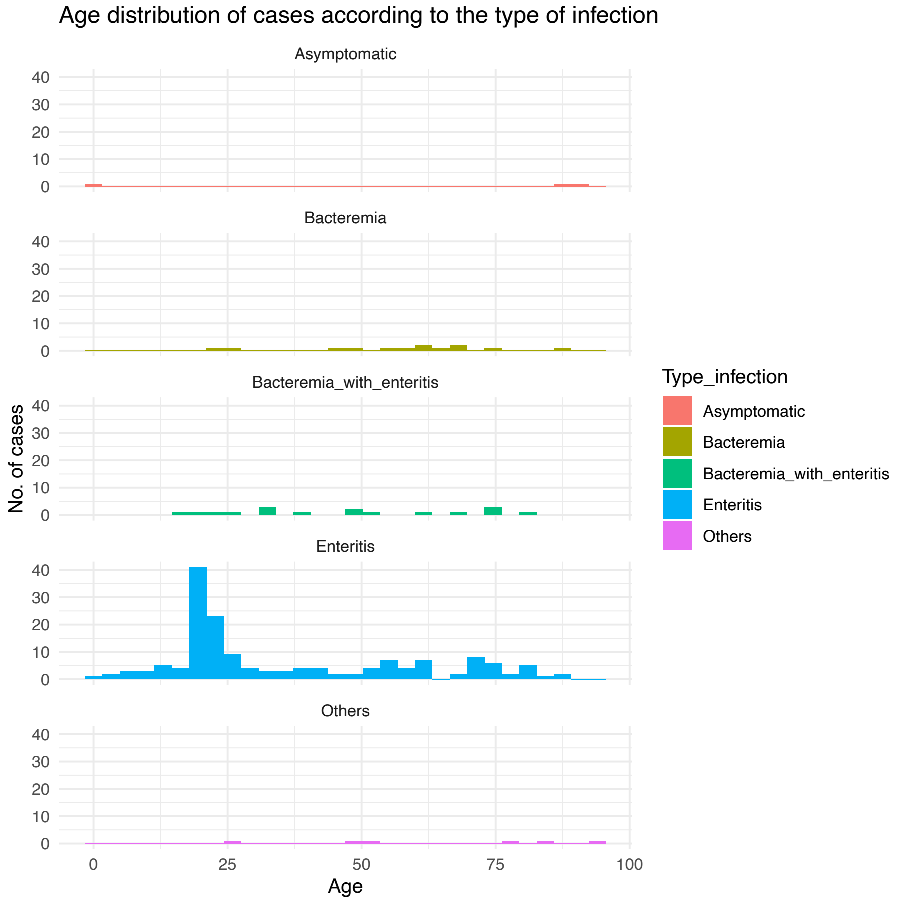

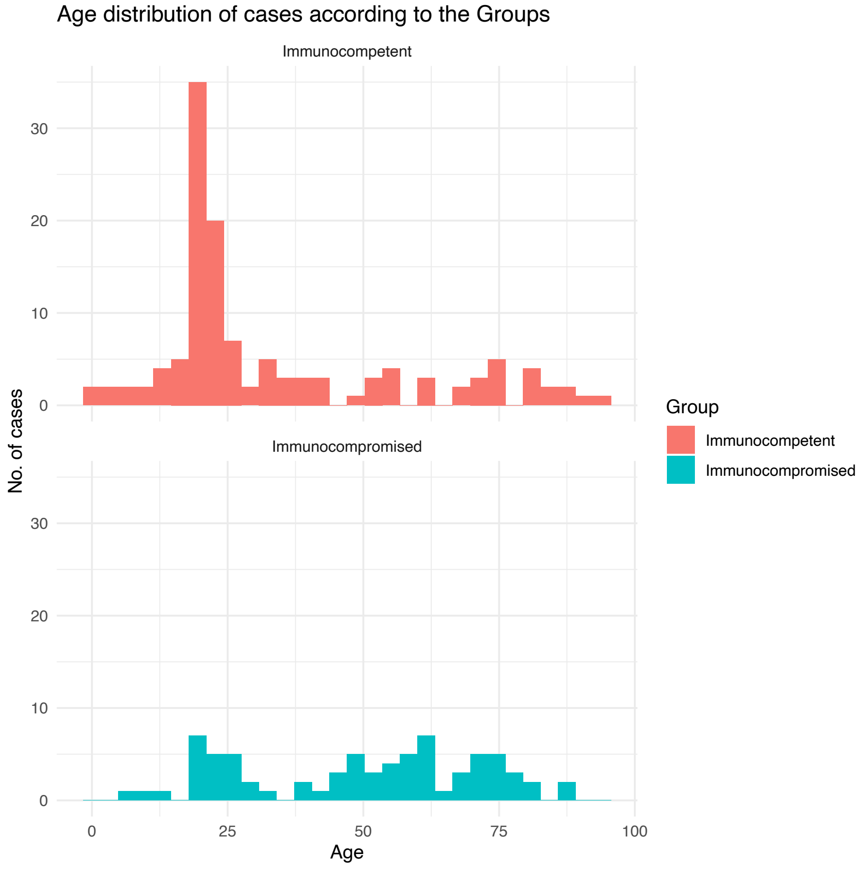

Supplement: ofaf406_Supplementary_Data [file ofaf406_supplementary_data.pdf]
